# Bridging the gap between computation and clinical biology: validation of cable theory in humans

**DOI:** 10.3389/fphys.2013.00213

**Published:** 2013-09-04

**Authors:** Malcolm C. Finlay, Lei Xu, Peter Taggart, Ben Hanson, Pier D. Lambiase

**Affiliations:** ^1^Department of Cardiac Electrophysiology, The Heart Hospital, Institute of Cardiovascular Science, University College LondonLondon, UK; ^2^Department of Mechanical Engineering, University College LondonLondon, UK

**Keywords:** conduction velocity restitution, computational modeling, action potential duration, patient specific modeling, cardiac arrhythmia

## Abstract

**Introduction:** Computerized simulations of cardiac activity have significantly contributed to our understanding of cardiac electrophysiology, but techniques of simulations based on patient-acquired data remain in their infancy. We sought to integrate data acquired from human electrophysiological studies into patient-specific models, and validated this approach by testing whether electrophysiological responses to sequential premature stimuli could be predicted in a quantitatively accurate manner.

**Methods:** Eleven patients with structurally normal hearts underwent electrophysiological studies. Semi-automated analysis was used to reconstruct activation and repolarization dynamics for each electrode. This *S*_2_ extrastimuli data was used to inform individualized models of cardiac conduction, including a novel derivation of conduction velocity restitution. Activation dynamics of multiple premature extrastimuli were then predicted from this model and compared against measured patient data as well as data derived from the ten-Tusscher cell-ionic model.

**Results**: Activation dynamics following a premature *S*_3_ were significantly different from those after an *S*_2_. Patient specific models demonstrated accurate prediction of the *S*_3_ activation wave, (Pearson's *R*^2^ = 0.90, median error 4%). Examination of the modeled conduction dynamics allowed inferences into the spatial dispersion of activation delay. Further validation was performed against data from the ten-Tusscher cell-ionic model, with our model accurately recapitulating predictions of repolarization times (*R*^2^ = 0.99).

**Conclusions:** Simulations based on clinically acquired data can be used to successfully predict complex activation patterns following sequential extrastimuli. Such modeling techniques may be useful as a method of incorporation of clinical data into predictive models.

## Introduction

Computerized simulations of cardiac activity have significantly contributed to our understanding of cardiac electrophysiology (Carusi et al., 2012). The recent leaps in raw processing power and access to supercomputing by research organizations has allowed proof-in-principle of many theories of cardiac conduction, from the cellular action potential to the generation of arrhythmia in heterogenous systems (e.g., in inherited arrhythmia syndromes such as Brugada or LQT syndromes). Though simulation has been very successful at bridging a knowledge gap between basic research findings and understanding of arrhythmia, efforts have principally concentrated on the “forward” solution, i.e., production of computer models that inform us of the behavior expected from our mathematical knowledge of cardiac physiology (Trayanova, 2011; Krummen et al., 2012). Few studies have attempted to broach a reverse solution, i.e., the construction of an accurate simulation based on patient-acquired data. There have been attempts to use patient anatomical data, such as those acquired from cardiac MRI, to fit a pre-existing cellular model, and some early work has attempted to fit clinically acquired data to a simulation (Relan et al., 2011). There remains some distance between computational modeling and clinical appreciation of arrhythmia generation, and computational approaches have not as yet found a clinical role.

Recently work has illustrated an approach by which electrophysiological data may be incorporated into simulations. Gilmour's group has concentrated on examination of one-dimensional conduction models, and has shown how sequential close-coupled activations may result in functional block “at-a-distance” from the stimulation site i.e., how an activating wavefront may impinge upon a prior wave of activation (Gilmour et al., 2007; Otani, 2007). Such functional block and wavebreak appear to be necessary events in triggering and sustaining the development of chaotic human arrhythmias, particularly VF. These frameworks incorporate both action potential duration (APD) restitution and conduction velocity restitution, and are founded on the cable theory principles originating in the work of Hodgkin and Huxley (Noble, 1962). In brief, this concept embodies cardiac conduction as a syncytium; strips of cardiac muscle are considered to act as uniform cables, allowing conduction in all directions. Unlike the squid giant axon, the cable properties of cardiac cells span many different cells, but they may still be represented as a two syncytial domains: intracellular and extracellular, and conduction properties depend on the properties of the cell membranes, ionic concentrations and cellular geometric arrangements. This concept can be developed to higher dimensions e.g., Spach et al., 1979, and/or simplified into descriptions of pure propagation of activation and repolarization e.g., Nolasco and Dahlen, 1968. These latter coupled-map models are easy to deal with both analytically and numerically and avoid the large complexities of high-dimensional, non-linear models of cardiac conduction. Further iterations of these models have clarified mathematical descriptions of conduction block at-a-distance in one-dimensional fiber (Fox, 2002; Fox et al., 2003).

Such a model underwent a qualitative validation in a canine model by Gelzer et al. (2008), where it was used to predict the initiation of VF by combinations of up to 5 extrastimuli. This work was notable in that the computer model had incorporated biologically-acquired data (action potential restitution measured from the interventricular septum). Thus, it represented a proof-of-concept of animal-specific electrophysiological models in the prediction of VF induction by extrastimuli, and has begun to bridge the gap between theoretical electrophysiological modeling and clinical application. But whether such models can be applied to the human heart, particularly in the investigation of cardiac risk, remains unproven.

A major limitation to closing the gap between ionic cardiac models and clinical implementation is the iterative process required to be undertaken to fit the model to the data (Clayton et al., 2011). The great number of variables in cardiac ionic models precludes accurately fitting of clinical data with confidence (Zaniboni et al., 2010). Previous attempts have concentrated on anatomical approaches to fit data to the individual, relying on “generic” models of the electrophysiology itself (Trayanova, 2011). Recognizing this, we have endeavored to describe observed cardiac electrophysiology at the tissue level using simple parameters.

This paper describes the approach we have taken to derive computer models from clinical data in our attempt to close the gap from theoretical modeling. The mathematical foundation of our work remains that of the one-dimensional model, which is itself a manifestation of cable theory. Thus, this work incorporating observed clinical parameters into a predictive model, and the validation of this method, in itself acts as a validation-step to the relevance of cable theory to human arrhythmogenisis.

We hypothesized that individual human restitution dynamics could be described by a one-dimensional model incorporating both conduction velocity restitution and action potential restitution. We tested this by incorporating patient-acquired data into individualized one-dimensional models (Hand and Griffith, 2010). These models were used to predict activation time (AT) dynamics following a second extrastimulus, and predictions were compared to experimental results.

## Methods

### Patient population and electrophysiological studies

**Figure 1:**
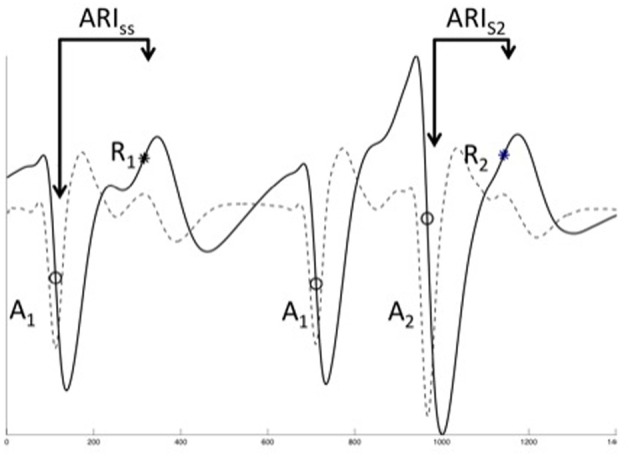
**Unipolar EGM analysis**. The three final of a restitution train are shown, typical unipolar electrograms (solid line) and their differential (dashed line). Local activation was defined as the most rapid downstroke (dVdtmin, circles, A_1_, A_2_) of the activation inscription, repolarization was defined using the classical (Wyatt) method as dVdtmax of the T-wave (asterisks, R_1_, R_2_). Activation time was defined as the time from pacing stimulus (not shown) to local activation, ARI as time from local activation to repolarization. It was not possible to reliably measure the diastolic interval for close coupled beats, therefore DI was taken as A_1_A_2_—ARI_*ss*_.

Studies were performed in patients undergoing cardiac electrophysiological studies with a view to diagnosis and ablation of supraventricular tachycardia. All patients gave prior informed consent. Studies were performed under minimal conscious sedation in the post-absorptive state. Antidysrhythmic drugs were discontinued for 5 days prior to the study. The study protocol had local ethics committee approval and conformed to the standards set by the Declaration of Helsinki. Decapolar catheters were placed in an epicardial coronary vein via the coronary sinus, at the RV apex and retrogradely within the LV cavity adjacent to the epicardial catheter (St Jude Pathfinder). A reference anodal electrode was placed in the inferior vena cava. Further catheters were placed according to clinical requirements (Figure 1). Electrograms were digitized and recorded at 1000 Hz (Bard Clearsign, MN, USA). If no arrhythmia was initiated by ventricular pacing during the initial clinical study, restitution studies were performed prior to further clinical testing.

### Study protocol

Following 3 min of steady-state pacing at the RV apex at a cycle length of 600 ms, a drivetrain of 10 beats was followed by an extrastimulus (*S*_2_). The *S*_1_*S*_2_ interval was decremented by 20 to 300 ms, thereafter in 5 ms intervals until ERP was reached. The ERP was found by increasing CIs by 8 ms and then decrementing by further 2 ms intervals. In patients undergoing second extrastimuli (*S*_3_) studies, the *S*_2_ extrastimulus was set at 10 ms above the observed refractory period, and an *S*_3_ was delivered at CIs progressively decrementing to a minimum of 180 ms.

### Waveform analysis

All electrogram analysis was performed using custom semi-automated software running in Matlab r2009b (The Mathworks Inc, MA, USA). Local AT and repolarization time (RT) were identified using an algorithm and manually checked. Filter settings of 0.1–150 Hz band-pass (for AT) and 40 Hz low-pass (RT) were used. Any electrogram in which the T-wave morphology was either inconsistent throughout the drivetrains or inadequately defined was excluded from analysis.

The electrograms of the ultimate 2 beats of each drive train and subsequent *S*_2_ were exported and analyzed. It is technically challenging to measure action potentials in humans at more than one site simultaneously. However, the activation-repolarization index (ARI), i.e., the time from local activation to local repolarization as calculated from a unipolar electrogram signal, is a well-validated surrogate for the APD. Techniques for derivation of ARI analysis from unipolar electrograms have been well-described (Franz et al., 1987; Yue et al., 2004; Hanson et al., 2009). Briefly, AT was defined at the steepest negative slope of the activation waveform (Figure 1), and local repolarization time defined as the steepest positive slope of the T-wave (Figure 1), as supported by in-depth analysis (Yue et al., 2004; Yue, 2006; Potse et al., 2007, 2009). The diastolic interval (DI) preceding *S*_2_ was calculated using measured values taken from steady state data. Similarly, the DI prior to a *S*_3_ beat was estimated using a restitution model previously obtained for each electrode location. AT and repolarization time (RT) were measured from the stimulus time (defined as *t* = 0). The ARI of each beat is defined as the time difference from AT to RT, such that the repolarization time RT = AT + ARI.

ARI was plotted against DI to give standard ARI restitution curves. AT was plotted against coupling interval to illustrate conduction delay. These graphs were used for model creation. All electrogram analysis was performed using custom semi-automated software running in Matlab r2009b (The Mathworks Inc, MA, USA). AT and RT were identified using an algorithm and manually checked. Filter settings of 0.1–150 Hz band-pass (for AT) and 40 Hz low-pass (RT) were used. Any electrogram in which the T-wave morphology was inconsistent throughout the drivetrains or inadequately defined was excluded from analysis.

### One-dimensional conduction model and CV restitution modeling

We derived a novel method whereby conduction velocity restitution can be quantified from biologically acquired data using a linear cell fiber model. Arbitrary length of cells of 0.1 mm was used for model derivation. Each cell within a fiber was assumed to have homogenous CV and APD restitution properties. Both of these factors were described by exponential functions.

If the length of a tissue unit (e.g., a myocyte) is assumed as Δ*x*, and the conduction velocity within tissue as CV, the AT and repolarization time of a cell *x*_*i*_ can be calculated as:

(1) $$\text{Activation time: }AT_{n}(x_{i})=AT_{n}(x_{i-1})+\frac{\Delta x}{CV_{n}(x_{i})}$$

(2) $$\text{Repolarization time: }RT_{n}(x_{i})=AT_{n}(x_{i})+ARI_{n}(x_{i})$$

Where: AT and RT are the AT and repolarization time; ARI is the activation recovery interval; *n* is the number of beat.

Hence, the AT and recovery time at cell *x*_*i*_ can be expressed as:

(3) $$AT_{n}(x_{i})=AT_{n}(x_{0})+\sum_{j=0}^{i}\frac{\Delta x}{CV_{n}(x_{j})}$$

(4) $$RT_{n}(x_{i})=AT_{n}(x_{0})+\sum_{j=0}^{i}\frac{\Delta x}{CV_{n}(x_{j})}+ARI_{n}(x_{i})$$

Where: *x*_0_ represents the site of stimulation.

Thus, the period between activation *n* and activation *n* + 1 for site *x*_*i*_ can be calculated as:

(5) $$\begin{array}{l} AT_{n+1}(x_{i})-AT_{n}(x_{i})=AT_{n+1}(x_{0})-AT_{n+1}(x_{0})\\ \;+\sum_{j=0}^{i}\left(\frac{\Delta x}{CV_{n+1}(x_{i})}-\frac{\Delta x}{CV_{n}(x_{i})}\right) \end{array}$$

Here, the period between activation *n* and activation *n* + 1 for site *x*_0_ equals to the coupling interval (*CI*_*n*+1_) applied at site *x*_0_

(6) $$AT_{n+1}(x_{0})-AT_{n}(x_{0})=CI_{n+1}$$

The period between activation *n* and activation *n* + 1 for site *x*_*i*_ can also be calculated as:

(7) $$AT_{n+1}(x_{i})-AT_{n}(x_{i})=ARI_{n}(x_{i})+DI_{n+1}(x_{i})$$

Combining equation 5, 6, and 7, the basic equation for one-dimensional conduction model can be expressed as:

(8) $$DI_{n+1}(x_{i})=CI_{n+1}+\sum_{j=0}^{i}\left(\frac{\Delta x}{CV_{n+1}(x_{j})}-\frac{\Delta x}{CV_{n}(x_{j})}\right)-ARI_{n}(x_{i})$$

**Figure 2:**
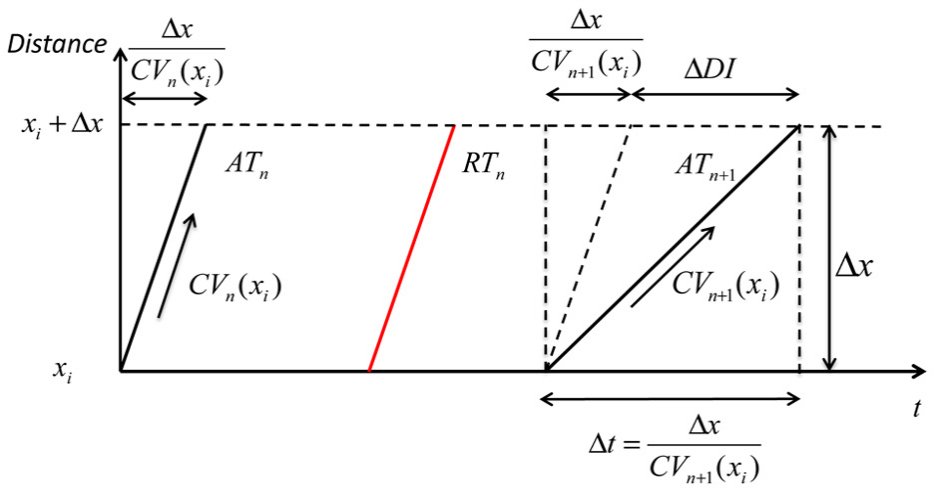
**Geometry relationship based on CV restitution**. A geometric relationship based on the CV restitution model is shown. *x*_*i*_ is the position along a conduction path, Δ*DI* is the change in diastolic interval, Δ*t* is the time that an activation wave takes to travel the distance Δ*x*. *AT*_*n*_: steady-state activation time with long DI; *RT*_*n*_: steady-state repolarization time; *AT*_*n*+1_: activation time of an early beat with shorter pacing interval and consequently showing some CV decrease (*CV*_*n*+1_ (*x*_*i*_), but as the distance over which this operates (Δ*x*) is very small this can be considered as a straight line.

The conduction velocity *CV*(*x*_*i*_) is assumed to be a function of its preceding DI:

(9) $$CV(x_{i})=CV_{SS}-B_{CV}\cdot e^{C_{CV}\cdot DI}$$

where *CV*_*SS*_ is the steady-state conduction velocity, a constant defined to be 0.72 (Fox, 2002); *B*_*CV*_ and *C*_*CV*_ are two constants determining the steepness of CV restitution curve. This derivation is summarized in Figure 2.

Referring to the experimental data, *DI*_*n*+1_ (*x*_*i*_) is the DI of premature beat *S*_2_, *ARI*_*n*_ is the ARI of the steady-state activation following *S*_1_ (*ARI*_*n*_ = *ARI*_*SS*_), *CI*_*n* + 1_ is the *S*_1_*S*_2_ coupling interval. These three parameters were directly obtained from experimental data. *CV*_*n*_ (*x*_*i*_) is the conduction velocity of a steady-state activation, i.e., is equal to *CV*_*SS*_.

For each investigated site, the activation conduction pathway from the pacing site to the investigated site was assumed to be an independent one-dimensional conduction pathway with uniform restitution properties. From the experimental data, an assumed length of the conduction pathway (given the assumed steady state conduction velocity) can be calculated by the product of steady-state conduction velocity and the steady-state conduction time, *AT*_*SS*_ (*x*_*i*_), from the pacing site to the investigated site:

(10) $$L(x_{i})=CV_{SS}\cdot AT_{SS}(x_{i})$$

Hence, the number of cells included in the conduction pathway is $i=\frac{L(x_{i})}{\Delta x}$.

Equation 8 can be rearranged into the following form, which allows its solution using experimental data:

(11) $$DI_{S2}(x_{i})+ARI_{SS}(x_{i})-CI_{S2}+AT_{SS}(x_{i})=\sum_{j=0}^{i}\frac{\Delta x}{CV_{S2}(x_{j})}$$

All the dynamics in the left hand side of equation 11 are those obtained from experimental data. For each investigated site, the one-dimensional conduction model and CV restitution model can be fitted with its dynamics from experimental data. An iterative approach was taken, adjusting *B*_*CV*_ and *C*_*CV*_ in equation 9, and a minimum least-squares error method was employed to select optimum parameters.

### Incorporation of patient data into one dimensional models

Data acquired from patient studies was applied to the model, and a separate (and independent) model was created for each electrode site. ARI restitution was modeled using a mono-exponential equation of the form specified in equation 13.

A standard value of 0.72 ms-1 was assumed as baseline conduction velocity, changing this absolute value by ±0.2 ms did not significantly affect derived CV dynamics. Despite model fitting being computationally intense, the use of simplified exponential dynamics allowed modern desktop machines to derive CV parameters reasonably quickly. For a typical patient, 100–2000 cells were included, depending on the distance of the conduction pathway. For example, if the steady-state conduction time was 100 ms at the investigated site, assuming the steady-state conduction velocity to be 0.72 ms-1 and cell length to be 100 μm, the number of cells included in the conduction model was 720.

Validation of our method was performed in two ways, comparison with ionic cellular model system and comparison with experimentally acquired patient data.

Once both ARI and CV restitution parameters had been derived from patient data, the cell fiber model was used to predict conduction dynamics of a second extrastimuli (*S*_3_) introduced following a short *S*_1_*S*_2_ coupling interval. Modeling of sequential beats was only attempted if the *r*^2^ for fitting to *S*_2_ data was >0.7 at that electrode site, and sites where electrograms were of poor quality were excluded. ATs following the *S*_3_ beat obtained experimentally were quantatively compared to those obtained from the cell fiber model.

### Comparing the exponential model of repolarization dynamics against data ionic cellular simulation

At short *S*_2_*S*_3_ coupling intervals, the QRS complex of the unipolar electrogram following an *S*_3_ can widen to fuse with the repolarization complex. This precludes using unipolar electrograms to validate our simple model for prediction of repolarization times. We thus performed preliminary validation of our model for prediction of *S*_3_ repolarization times against data taken from an ionic cellular model. The ion channel model is obtained from ten Tusscher et al. from their studies of human ventricular tissue (ten Tusscher et al., 2009).

The ten Tusscher human ventricular ionic model includes 16 types of currents, and 30 ionic gate variables. It is practically impossible with current technology to obtain detailed information of each type of ion current or gate variables on an individual patient. However, the experimental data provides information on key cellular properties such as APD, AT, and repolarization time. These three interdependent properties were fitted with the restitution models, which is then used to simulate interactions of activation and repolarization.

The ten Tusscher human ventricular model can be summarized in a general form below (the forward Euler method was used to integrate the model). Initial conditions for ionic values and conductances are given in Table 1:

(12) $$\frac{\partial V}{\partial t}=-\frac{I_{ion}+I_{stim}}{C_{m}}$$

Where: *V* is voltage,

*t* is time,

*I*_*ion*_ is the sum of all transmembrane ionic currents,

*I*_*stim*_ is the stimulus current,

*C*_*m*_ is cell capacitance per unit surface area.

**Table 1:** **Initial conditions for state variables in human ventricular ionic model**.

*V*	−87.84 mV	[*Na*^+^]_*i*_	7.23 mM	[*Na*^+^]_*ss*_	7.23 mM
[*K*^+^]_*i*_	143.79 mM	[*K*^+^]_*ss*_	143.79 mM	[*Ca*^2+^]_*i*_	8.54•10^−5^ mM
[*Ca*^2+^]_*ss*_	8.43•10^−5^ mM	[*Ca*^2+^]_*nsr*_	1.61 mM	[*Ca*^2+^]_*jsr*_	1.56 mM
*m*	0.0074621	*h*_*fast*_	0.692591	*h*_*slow*_	0.692574
*j*	0.692574	*h*_*CaMK,slow*_	0.448501	*j*_*CaMK*_	0.692413
*m*_*L*_	0.000194015	*h*_*L*_	0.496116	*h*_*L,CaMK*_	0.265885
*a*	0.00101185	*i*_*fast*_	0.999542	*i*_*slow*_	0.589579
*a*_*CaMK*_	0.000515567	*i*_*CaMK,fast*_	0.999542	*i*_*CaMK,slow*_	0.641861
*d*	2.43015•10^−9^	*f*_*fast*_	1	*f*_*slow*_	0.910671
*f*_*Ca,fast*_	1	*f*_*Ca,slow*_	0.99982	*j*_*Ca*_	0.999977
*n*	0.00267171	*f*_*CaMK,fast*_	1	*f*_*Ca,CaMK,fast*_	1
*x*_*r,fast*_	8.26608•10^−6^	*x*_*r,slow*_	0.453268	*x*_*s*1_	0.270492
*x*_*s*2_	0.0001963	*x*_*K*1_	0.996801	*J*_*rel,NP*_	2.53943•10^−5^mM/ms
*J*_*rel,CaMK*_	3.17262•10^−7^mM/ms	*CaMK*_*trap*_	0.0124065		

Where: V, membrane voltage; [Na^+^]_i_, intracellular sodium ion concentration; [Na^+^]_ss_, concentration of sodium ion, in subspace compartment; [K^+^]_i_, intracellular potassium ion concentration; [K^+^]_ss_, concentration of potassium ion, in subspace compartment; [Ca^2+^]_i_, intracellular calcium ion concentration; [Ca^2+^]_ss_, concentration of calcium ion, in subspace compartment; [Ca^2+^]_nsr_, concentration of calcium ion, in network SR compartment; [Ca^2+^]_jsr_, concentration of calcium ion, in junctional SR compartment; m, activation gate for fast Na^+^ current I_Na_; h_fast_, fast development of inactivation gate for fast Na^+^ current I_Na_; h_slow_, slow development of inactivation gate for fast Na^+^ current I_Na_; j, recovery from inactivation gate for fast Na^+^ current I_Na_; h_CaMK,slow_, slow development of inactivation gate for CaMK phosphorylated fast Na^+^ current I_Na_; j_CaMK_, recovery from inactivation gate for CaMK phosphorylated fast Na^+^ current I_Na_; m_L_, activation gate for late Na^+^ current I_Na_; h_L_, inactivation gate for late Na^+^ current I_Na_; h_L,CaMK_, inactivation gate for CaMK phosphorylated Na^+^ current I_Na_; a, activation gate for transient outward K^+^ current I_to_; i_fast_, fast inactivation gate for transient outward K^+^ current I_to_; i_slow_, slow inactivation gate for transient outward K^+^ current I_to_; a_CaMK_, activation gate for CaMK phosphorylated transient outward K^+^ current I_to_; i_CaMK,fast_, fast inactivation gate for CaMK phosphorylated transient outward K^+^ current I_to_; i_CaMK,slow_, slow inactivation gate for CaMK phosphorylated transient outward K^+^ current I_to_; d, activation gate for Ca^2+^ current through the L-type Ca^2+^ channel I_CaL_; f_fast_, fast voltage dependent inactivation gate for Ca^2+^ current through the L-type Ca^2+^ channel I_CaL_; f_slow_, slow voltage dependent inactivation gate for Ca^2+^ current through the L-type Ca^2+^ channel I_CaL_; f_Ca,fast_, fast development of Ca^2+^ dependent inactivation gate for Ca^2+^ current through the L-type Ca^2+^ channel I_CaL_; f_Ca,slow_, slow development of Ca^2+^ dependent inactivation gate for Ca^2+^ current through the L-type Ca^2+^ channel I_CaL_; j_Ca_, recovery from Ca^2+^ dependent inactivation gate for Ca^2+^ current through the L-type Ca^2+^ channel I_CaL_; n, fraction in Ca^2+^ dependent inactivation mode for Ca^2+^ current through the L-type Ca^2+^ channel I_CaL_; f_CaMK,fast_, fast development of Ca^2+^ dependent inactivation gate for CaMK phosphorylated I_CaL_; f_Ca,CaMK,fast_, slow development of Ca^2+^ dependent inactivation gate for CaMK phosphorylated I_CaL_; x_r,fast_, fast activation/deactivation gate for rapid delayed rectifier K^+^ current I_Kr_; x_r,slow_, slow activation/deactivation gate for rapid delayed rectifier K^+^ current I_Kr_; x_s1_, activation gate for slow delayed rectifier K^+^ current I_Ks_; x_s2_, deactivation gate for slow delayed rectifier K^+^ current I_Ks_; x_K1_, inactivation gate for inward rectifier K^+^ current I_K1_; J_rel,NP_, non-phosphorylated Ca^2+^ release, via ryanodine receptors, from jsr to myoplasm; J_rel,CaMK_, CaMK phosphorylated Ca^2+^ release, via ryanodine receptors, from jsr to myoplasm; CaMK_trap_, fraction of autonomous CaMK binding sites with trapped calmodulin.

Action potential duration (APD) is defined to be action potential duration at 90% repolarization (APD_90_).

Diastolic interval (DI) equals to the coupling interval of S2 minus APD from steady-state beat (APD_*ss*_).

The form of exponential model tested against data acquired from the ionic cellular model was:

(13) $$APD=APD_{ss-B•e^{C•DI}}$$

The pacing protocols applied to ion channel model were the same as those used clinically (i.e., a sequential extrastimulus study). Data acquired from the ion channel model was compared against *S*_3_ repolarization times modeled using our simple exponential-based approach.

### Statistics

Data is presented as mean ± standard deviation unless otherwise stated. Comparative statistics were calculated within R. An individual simulation was performed for each measured site, thus each patient had multiple simulations performed. Multilevel regression enabled appreciation of the statistical reliability of predictions without inappropriate prejudice or favor regarding the grouped and leveled nature of measurements (i.e., incorporating both within-subject and between-subject variances). These calculations were performed using the lmer package (Pinheiro et al., 2009), single-level comparisons of normally-distributed continuous variables were performed using Student's *T*-test. A *p*-value of less than 0.05 was taken as significant. Correlation coefficients involving model comparisons were performed with Matlab.

## Results

### Patient demographics

Eleven patients were enrolled in the study, aged 29 ± 10 years, 7 females. All patients had structurally normal hearts on echocardiography, and underwent EP studies for symptoms consistent with paroxysmal supraventricular tachycardia. Final diagnoses were atrioventricular re-entrant tachycardia (5 patients), paroxysmal atrial fibrillation (1). No arrhythmia was induced in 5 patients. Six of these patients underwent *S*_3_ studies and were included in the *S*_3_ validation.

### Activation time, ARI restitution and repolarization curves

**Figure 3:**
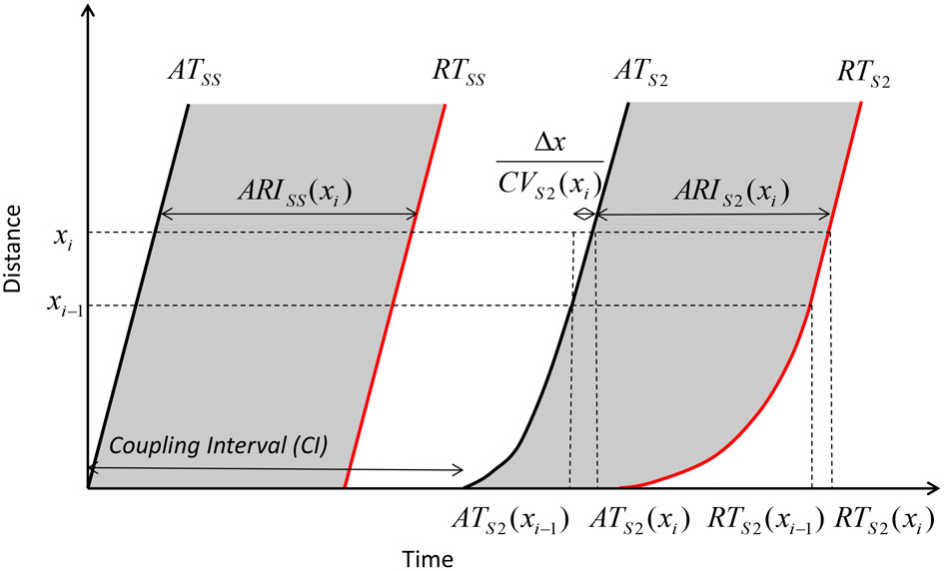
**Geometry relationship and derivation of modulation of dispersion**. A premature extrastimulus results in conduction velocity restitution. Consequently, ATs are delayed, but this delay allows resumption toward steady state conduction velocities over the course of the activation path. The modulation of activation dispersion is amplified via ARI restitution on the repolarization wavefront, and dispersion of repolarization increases further.

**Figure 4:**
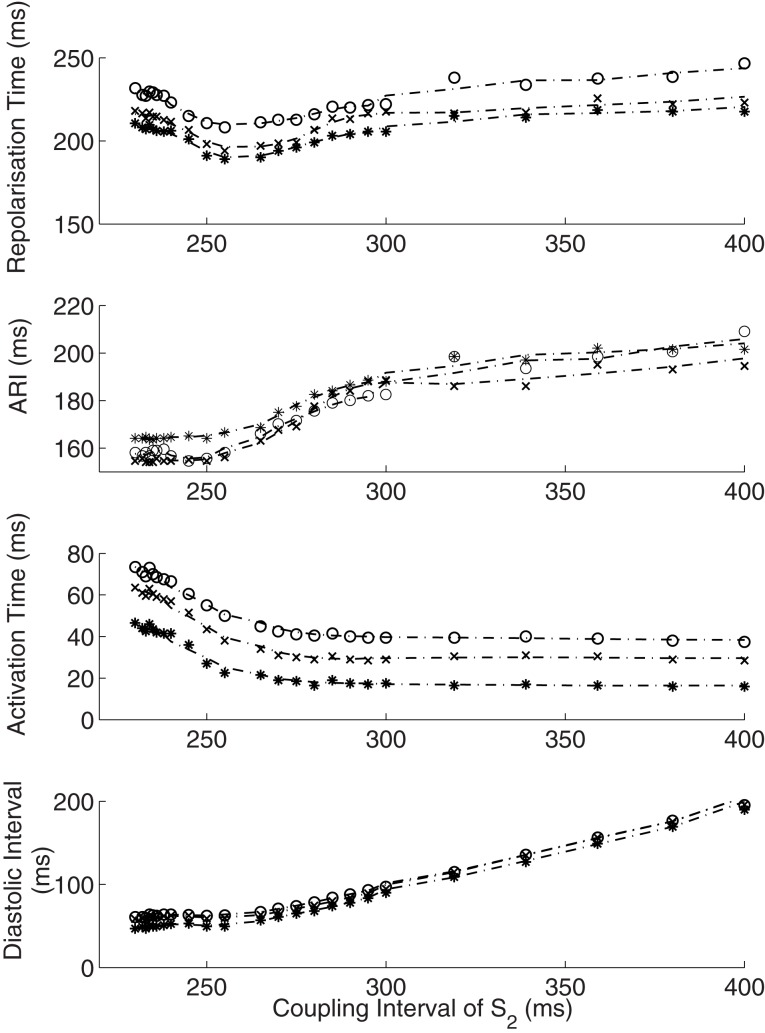
**Interactions of activation and ARI leading to repolarization time change**. The response of diastolic interval (DI), activation time and ARI to shortening coupling interval are shown, with the overall repolarization time dynamic shown in the top panel. As paced intervals decrease, diastolic intervals decrease initially in a linear fashion (**Bottom** panel). The reduction in DI is associated with a non-linear reduction in ARI (restitution). As coupling intervals become very short, activation time increases due to the effect of conduction velocity restitution acting between the stimulus site and the recording electrode. Activation time increases prevent further reduction of local diastolic interval (**Bottom** panel), which is seen to level off. ARI restitution is blunted at these short coupling intervals. Repolarization time thus increases as a result of increasing activation times. Representative patient data from RV (Star—early activating site, cross- mid activating site, circle- late activating site, dotted lines represent smoothed dynamics).

The dynamics of how AT, ARI, repolarization time (RT) and DI varied with the coupling interval of the first extrastimulus were examined. This is illustrated in Figure 3. As coupling interval decreases from steady state, ARI restitution is observed, and both ARI and repolarization time shorten. But as coupling intervals shortened further toward ERP, the effects of conduction velocity restitution became apparent (as illustrated by increasing ATs). This prevents further decrease in local DIs, and hence ARI restitution is restricted. The local repolarization time effectively represents the cumulative effect of ARI and conduction velocity restitution, together these produced a distinctive repolarization time curve illustrated in Figure 4.

### Model fitting to *S*_2_ data

One-dimensional models were fitted to clinically acquired *S*_2_ data in all 11 patients. ARI restitution curves were accurately described by a simple exponential model (Coefficient of determination, *r*^2^ = 0.966 [95% CI 0.963–0.968], *p* < 0.0001). AT curves were similarly accurately reproduced following our derivations of conduction velocity restitution, (*r*^2^ = 0.993 [0.992–0.993], *p* < 0.0001).

### Activation times following sequential premature stimuli

To show that prediction of ATs following sequential extrastimuli was a reasonable standard by which to assess the accuracy of our model, we compared the activation dynamics following *S*_2_ and *S*_3_ stimuli. At similar coupling intervals, AT following a premature *S*_3_ was significantly shorter than after an *S*_2_ [AT normalized to steady state: 1.1 following *S*_3_ (95% confidence interval 1.1–1.2) vs. 1.6 following *S*_2_ (1.5–1.7), *p* < 0.0001]. ERP of S_3_ was shorter than ERP of *S*_2_ (<186 ± 8 vs. 222 ± 25 ms, *p* < 0.0001). More activation delay existed after an S_3_ pre-ERP than after an *S*_2_ (39 ± 28 vs. 33 ± 16 ms, *p* = 0.0003). Activation dynamics were thus significantly different following *S*_2_ and *S*_3_ stimuli, with the ERP of an *S*_2_ considerably shorter than that following the steady state beats.

### Validation of 1D models

**Figure 5:**
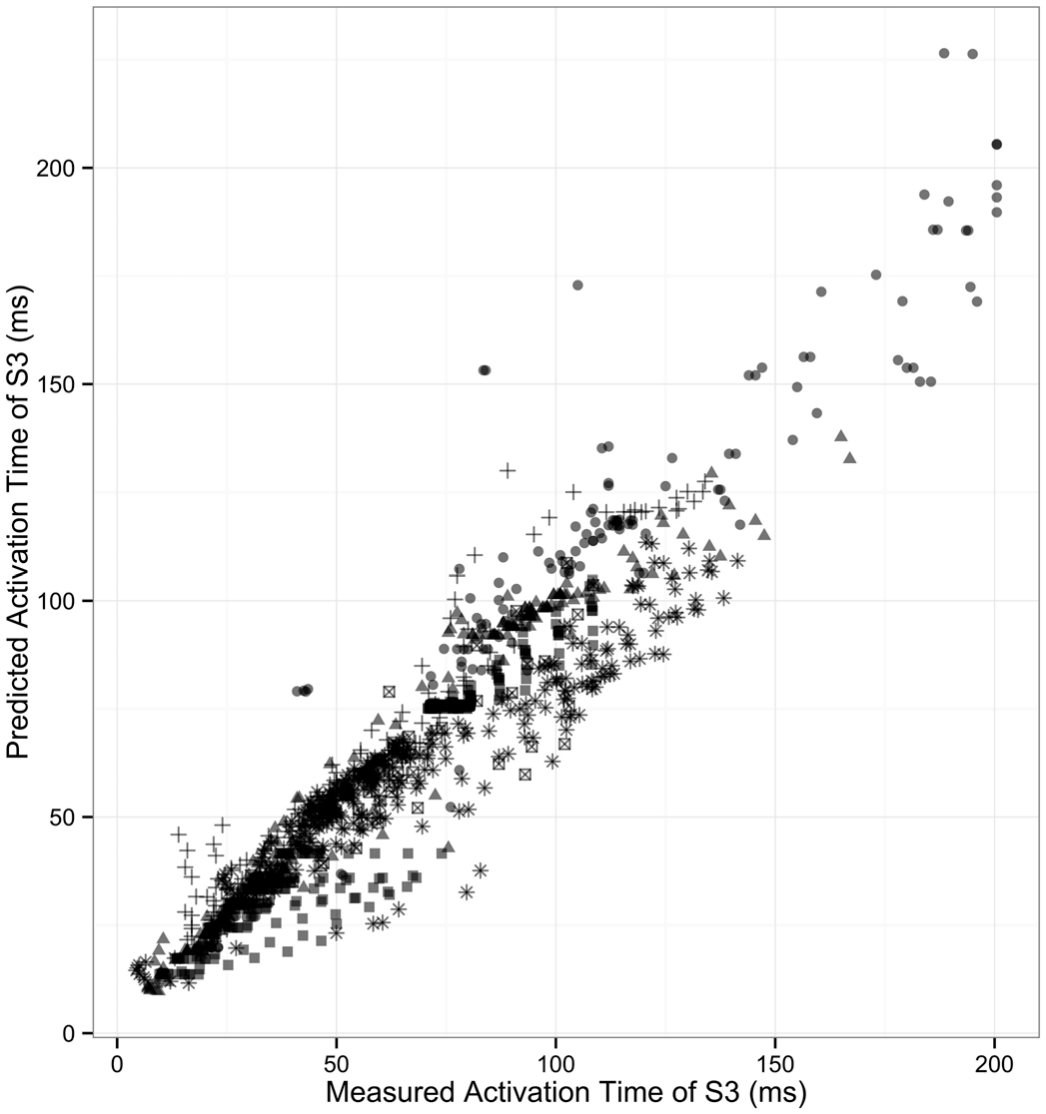
**Correlation of measured and predicted AT of second extrastimulus**. The correlation of modeled S_3_ activation time with measured S_3_ activation is shown. Each patient is represented by individual symbols. *r*^2^ = 0.90.

We tested whether the one-dimensional model, incorporating both ARI and CV restitution, could explain and predict the observed variation in activation dynamics following *S*_2_ and *S*_3_ stimuli. This serves as a quantative validation of the 1D model incorporating CV and ARI restitution. Validation was performed using data from 6 patients who underwent *S*_3_ protocol. The coefficient of determination (predicted values vs. measured), *R*^2^, was 0.902 [95% CI: 0.890–0.911] (*p* < 0.0001, Figure 5). The high predictive accuracy possible with this method serves as a quantitative validation of this one-dimensional model of cardiac activation in human hearts.

### Simulating modulation of dispersion of repolarization

**Figure 6:**
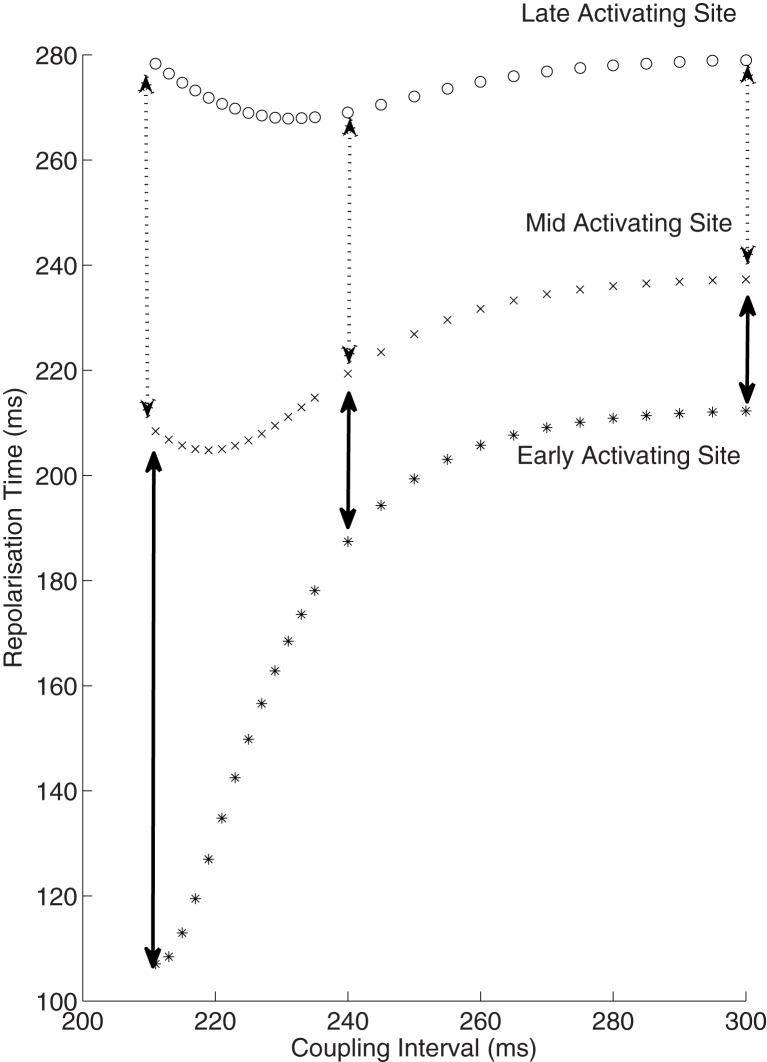
**Increase in dispersion of repolarization with reducing coupling intervals**. Repolarization times from 3 sites are shown against coupling intervals. The early activating site can be considered to be equivalent to the stimulus site, and is not measured clinically due to artifact. This early site is exposed to only minimal activation time increase at short coupling intervals, thus the repolarization time is approximately equal to the ARI at this site. The mid and late activating sites do exhibit an increase in dispersion of repolarization (broken arrows), but this is minor in comparison to the increase from the early to mid site (solid arrows). Thus, there is a marked spatial increase in the repolarization gradient near the stimulus site following premature extrastimuli.

Simulations based on patient data enable examination of electrophysiology which otherwise remains impenetrable through traditional experimental methods. We examined how dispersion of repolarization varied with shortening of *S*_2_ coupling interval. True dispersion of repolarization, the difference from the earliest repolarization to the latest repolarization, has both a spatial and temporal component. It is very hard to measure repolarization time accurately very close to the stimulus site due to stimulus artifact. But the most significant repolarization gradients will be close to the stimulus site following a close-coupled *S*_2_; the area immediately at the stimulus site is exposed to very little delay prior to activation, whereas further sites only a very short distance away experience significant activation delay. A patient-based simulation allows appreciation of these gradients (Figure 6). In the example shown the dispersion of repolarization increased from 41 to 70 ms between mid to late activating sites, yet increased 4-fold from 25 to 100 ms between earlier activating sites. Thus, the induction of repolarization gradients by premature stimuli occurs very close to the stimulus site.

### Validation of repolarization time predictions against ionic cellular simulations

For *S*_3_ responses, there was close agreement between repolarization times determined from our model and repolarization times acquired using the ionic model (coefficient of determination, *r*^2^ = 0.997 [95% CI 0.993–0.999], *p* < 0.0001). This supports the validity of our model in comparison to full ionic cellular models undergoing sequential premature extrastimuli.

## Discussion

Using a combination of physiological measurement and simulation, this study demonstrates that the complex dynamic interactions between activation and repolarization induced by sequential extrastimuli can approximated in terms of ARI (APD) and CV restitution. This serves as a strong validation of a personalized computational approach incorporating biologically acquired variables and it serves as an extension and validation in humans of previous animal work (Gilmour et al., 2007). Our technique provides a platform by which individual patient data may be incorporated into computerized simulations and serves as an example of a method by which the gap between fundamental cardiac simulation and clinical science can be bridged.

This study is the first to quantitatively validate a one-dimensional model of cardiac conduction in man. Such patient-based models provide a method by which physiologically acquired data may be interrogated in a computerized simulation. We have shown that endocardial physiology can be adequately modeled by simple mathematical descriptors of CV and ARI restitution curves, and confirm the relevance of one-dimensional conduction models incorporating both these features to human cardiac electrophysiology. Our validation of a straightforward one-dimension model can allow further in-depth examination of human physiology in a manner that would not be possible experimentally, for both ethical and practical reasons.

The presented modeling is founded on the work of Otani, Gilmour and Fox (Fox et al., 2003; Gilmour et al., 2007; Otani, 2007; Gelzer et al., 2008, 2010). Seminal work by this group, along with the single fiber models of Cherry and Fenton (2004, 2007), provided initial validation of the use of one-dimensional conduction simulation in biological systems. The ability of such a model to predict whether large variations of long-short CIs would induce VF in normal and cardiomyopathic canines was a powerful demonstration of the physiological relevance of such simulations (Gelzer et al., 2008, 2010).

We have enhanced these models by fully deriving CV restitution; allowing better approximation to the actual behavior of the activation wavefront and observation of the heterogeneity introduced by sequential extrastimuli. This adds to our earlier description of the modulated dispersion of repolarization in humans (Hanson et al., 2009), and addresses questions raised from this work (Poelzing and Rosenbaum, 2009). CV restitution is often assumed to operate over the entire conduction path in a similar manner, obscuring the importance of the spatial differences in CV restitution. Our validation also sought a high fidelity correlation of ATs rather than a binary outcome. The large datasets acquired from the multiple recording electrodes used in each patient (rather than only one in the canine studies) enabled an accurate validation of this concept in humans.

The degree of latency has been described as being important in determining the initiation of VT or VF due to programmed electrical stimulation in earlier clinical studies (Avitall et al., 1992), yet the mechanism of this has been unclear. Our explanation of CV restitution, and of a mechanism by which cumulative interactions can set up conduction heterogeneities is consistent with these previous descriptions.

Knowledge of these timing dynamics may help determine susceptibility to arrhythmia in channelopathies (Lambiase et al., 2009; Nam et al., 2010) or a suspected cardiomyopathy. The demonstration that patient-specific modeling can predict initiation sites of clinical arrhythmia underlines the importance of ARI and CV interactions in human arrhythmogenisis.

### Study limitations

We used clinically acquired data to observe and explain the complex interactions seen following sequential extrastimuli. These are considered central to the initiation of cardiac arrhythmia. We did not expect to, nor did we induce arrhythmia in any patient undergoing prospective evaluation, and the contribution of these mechanisms in clinical arrhythmogenisis was not fully examined. Prospective patient data was acquired from contact electrodes; simultaneous mapping from the entire endo- and epicardial surface is unfeasible in conscious patients. However, as a result of lack of 3D geometry, conduction path distances were derived from timing data and knowledge of electrode spacings. The demonstration that these techniques can effectively predict wavebreak and arrhythmia initiation requires confirmation in prospective studies.

By design, our modeling and simulation was relatively simple and was limited to a one-dimensional system. This did not allow examination of wavebreak, arrhythmia development or re-entry, and observations are limited to assumptions of homogenous properties. The APD and CV restitution models used in this paper are in the form of exponential equations, this assumed behavior is based on the tendency of experimental data and on equations used in previous studies. Biological behavior would be expected to deviate from these simple exponential models, which in turn will result in discrepancies between simulation and clinical measurement. The use of derivations allowing direct fitting of ionic models to clinical results, perhaps by using an iterative approach, will improve the fidelity of such patient-derived simulations.

The success of our model at predicting local ATs following an *S*_3_ stimulus makes it tempting to assume that it will retain predictive accuracy for *S*_4_, *S*_5_ and so on. This is unlikely. Though the principles demonstrated remain valid, our model did not include myocardial memory effects, post-repolarization refractoriness or anisotropy. These may have increasing importance with longer extrastimuli sequences, and ARI restitution dynamics may change significantly as a consequence of these (Kobayashi et al., 1992; Shimizu et al., 2000). Despite a very high agreement in predicting *S*_3_ ARI restitution obtained from a complex ionic model, even these ionic models may still not fully reproduce these less studied phenomena. However, our model's simplicity made it accessible to the incorporation of clinical data, and the robust prediction and demonstration of *S*_3_ wavefront dynamics (which do not require knowledge of *S*_3_ ARI restitution) remains impressive.

### Future studies

This study provides important information to assist electrophysiological characterization of myocardial diseases and phenotypes. Some of the techniques we describe may be applicable to clinical practice e.g., determining an individual patient heart's boundary conditions for functional block as a method for arrhythmic risk stratification. All data was acquired in patients with normal hearts under baseline conditions. Further studies should examine the modulation of changes in conduction/repolarization interactions under conditions of autonomic stress or drug interactions. More complicated models, such as those based on ionic cellular models could be applied to the data, possibly using our method as an intermediary step to allow parameter fitting. This could produce more detailed and realistic simulations, extending the described parameters into 3 dimensions. Furthermore, new technologies such as non-invasive body surface mapping may allow acquisition of whole heart activation and repolarization dynamic data.

Specific future uses of the described method may have an early application in patients at risk of inherited ventricular arrhythmias, particularly Brugada syndrome or arrhythmogenic right ventricular cardiomyopathy (ARVC). Both of these conditions have well-described changes in invasive electrophysiology, yet risk stratification is incomplete. Patient specific models could give valuable diagnostic information and help quantify the risk of arrhythmia in such patients. Pharmaceutical testing may also benefit from such analysis, as a method to determine arrhythmogenicity of subtle changes in electrophysiological responses after drug challenge. Furthermore, a bullish use of this method would be as an adjunct in early clinical assessments of new anti-arrhythmia agents, to lend support to an investigational compound's antiarrhythmic properties, or otherwise.

## Conclusions

Conduction and repolarization interactions in humans can be described in terms of restitution of CV and APD. Simulations based on clinically acquired data can be used to successfully predict complex activation patterns following sequential extrastimuli. Such modeling techniques may be useful as a method of incorporation of clinical data into predictive models.

### Conflict of interest statement

The authors declare that the research was conducted in the absence of any commercial or financial relationships that could be construed as a potential conflict of interest.

## Abbreviations

ARI: Activation Recovery Interval
APD: Action potential duration
CV: Conduction Velocity
AT: Activation Time
ECG: Electrocardiogram
S1, S2, S3: Stimulus, Extrastimulus, Second Extrastimulus
ERP: Effective Refractory Period
RT: Repolarization Time
MRI: Magnetic Resonance Imaging
VT: Ventricular Tachycardia
VF: Ventricular Fibrillation.
